# Supplementary material for: Deliberative panels as a source of public knowledge: A large-sample test of the Citizens’ Initiative Review
Source: PLoS One. 2023 Jul 27;18(7):e0288188. doi: 10.1371/journal.pone.0288188 (PMC10374050; doi:10.1371/journal.pone.0288188)
Supplement: S1 File — (DOCX) [file pone.0288188.s001.docx]

# Online Supplement: Tables and Figures

# Table S1: Data Collection Details

| **Year** | **Election** | **Election Date** | **Ballot Measure** | **Issue** | **Mode and Survey Dates** | **Full *N*** |
| --- | --- | --- | --- | --- | --- | --- |
| 2010 | Oregon General | 2-Nov | Measure 73 | Sentencing | Online Oct 22-Nov 1 | 971 |
| 2012 | Oregon General | 6-Nov | Measure 85 | Kicker | Online Oct 4-Nov 5 | 1,539 |
| 2012 | Oregon General | 6-Nov | Measure 82 | Casinos | Online Oct 4-Nov 4 | 159* |
| 2014 | Jackson County Local | 20-May | Measure 15-119 | GMO Seeds | Mail May 10-Apr 9 | 4,600 |
| 2014 | Oregon General | 4-Nov | Measure 90 | Top-Two Primary | Online Oct 16-Nov 7 | 2,077 |
| 2014 | Oregon General | 4-Nov | Measure 92 | GMO Labels - OR | Online Oct 16-Nov 7 | 2,077 |
| 2014 | Colorado General | 4-Nov | Proposition 105 | GMO Labels - CO | Online Oct 16-27 | 1,816 |
| 2016 | Arizona General | 4-Nov | Proposition 205 | Marijuana - AZ | Online Oct 14-Nov 4 | 2,219 |
| 2016 | Oregon General | 8-Nov | Measure 97 | Gross Receipts - OR | Online Oct 14-Nov 8 | 2,100 |
| 2016 | Mass. General | 8-Nov | Question 4 | Marijuana - MA | Online Oct 14-Nov 1 | 2,250 |
| 2018 | Portland MSA | 8-Nov | Bond Measure | Housing - PDX | Online October 11-Nov 5 | 800 |
| 2018 | Mass. General | 6-Nov | Question 1 | Nursing - MA | Online October 11-Nov 5 | 1,600 |
| 2018 | Calif. General | 6-Nov | Proposition 10 | Housing - CA | Online October 11-28 | 800 |

Notes. Full *N* sample sizes include every respondent surveyed, even if not included in the experiments presented herein.

* The survey on this ballot measure was scaled back when the proponents suspended their campaign, essentially conceding the election, shortly after the survey launched.

# Table S2: Multilevel Modeling Factual Accuracy

*Note:* Multilevel ordered logit regression. Robust standard errors.

# Table S3: Pooled Factual Accuracy (Percent)

|  | **Not Shown CIR Statement** | **Shown CIR Statement** |
| --- | --- | --- |
| **Confidently Incorrect** | 9.2 | 6.8 |
| **Incorrect** | 21.5 | 15.3 |
| **Unsure/Don’t Know** | 24.8 | 20.4 |
| **Correct** | 28.3 | 30.8 |
| **Confidently Correct** | 16.4 | 26.7 |

*Note:* Data from all studies, independent of experimental design.

# Figure S1: Policy Knowledge Gains from Reading a CIR Statement

*Note*: Pooled tabulations.

# Table S4: Multilevel Modeling Factual Accuracy (Percent)

|  | **Not Shown CIR Statement** | **Shown CIR Statement** |
| --- | --- | --- |
| **Confidently Incorrect** | 9.1 | 6.5 |
| **Incorrect** | 21.2 | 17.0 |
| **Unsure/Don’t Know** | 24.7 | 23.0 |
| **Correct** | 27.8 | 30.6 |
| **Confidently Correct** | 17.4 | 22.9 |

*Note:* Data from all studies, independent of experimental design. Controlling for Issues and Claims across studies

# Figure S2: Knowledge Gains Across Studies


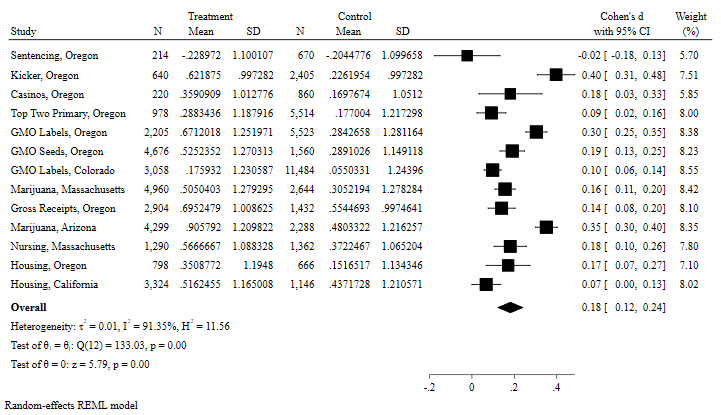


*Note:* This random-effects REML model’s forest plot provides more detail to the one presented in the main paper as Figure 2. N refers to number of Factual Accuracy observations, i.e. number of respondents times the number of factual items surveyed.

# Table S5: Holm Multiple Hypotheses Correction

| **Knowledge Item** | **Original *p*-value** | **Holm Corrected *p*-value** |
| --- | --- | --- |
| 108 | 0.000000000000000000634 | 0.000000000000000043746 |
| 84 | 0.00000000000000417 | 0.00000000000028356 |
| 109 | 0.0000000000000727 | 0.0000000000048709 |
| 94 | 0.000000000000243 | 0.000000000016038 |
| 101 | 0.00000000000418 | 0.0000000002717 |
| 106 | 0.00000000433 | 0.00000027712 |
| 77 | 0.0000000131 | 0.0000008253 |
| 107 | 0.000000237 | 0.000014694 |
| 52 | 0.000000812 | 0.000049532 |
| 113 | 0.00000133 | 0.0000798 |
| 50 | 0.00000208 | 0.00012272 |
| 100 | 0.0000055 | 0.000319 |
| 49 | 0.00000649 | 0.00036993 |
| 76 | 0.00000696 | 0.00038976 |
| 110 | 0.000009 | 0.000495 |
| 45 | 0.0000144 | 0.0007776 |
| 51 | 0.000019 | 0.001007 |
| 126 | 0.00003 | 0.00156 |
| 47 | 0.0000814 | 0.0041514 |
| 85 | 0.0001071 | 0.005355 |
| 130 | 0.0001369 | 0.0067081 |
| 104 | 0.0011617 | 0.0557616 |

*Note:* Table truncated after reaching .05 significance-level threshold.

# Table S6: Reading the CIR Statement Compared to Reading the Voter Guide

*Note:* Multilevel ordered logit regression. Robust standard errors.

# Figure S3: Policy Knowledge Gains – Reading the CIR Statement

Compared to Reading the Voter Guide

*Note*: Estimates from multilevel modeling.

# Table S7: Reading the CIR Statement and Voter Guide Compared to Only Reading the Voter Guide

*Note:* Multilevel ordered logit regression. Robust standard errors.

# Table S8: Reading the CIR Statement Compared to No Exposure

*Note:* Multilevel ordered logit regression. Robust standard errors.

# Table S9: Mean Difference in Factual Accuracy in Control Group (No Exposure)

*Note*: Welch’s t-test. Democrats = 0; Republicans = 1.

# Table S10: Interaction Model Testing Difference in Treatment Effect

*Note:* Multilevel ordered logit regression. Interaction between exposure to CIR and political party identification (Democrats reference group). Robust standard errors.

# Table S11: Policy Knowledge Gains and Faith in Deliberations (Scale)

*Note:* Multilevel ordered logit regression. Interaction between exposure to CIR Guide and Faith in Deliberation scale. Robust standard errors.
