# Supplementary material for: Deliberative panels as a source of public knowledge: A large-sample test of the Citizens’ Initiative Review
Source: PLoS One. 2023 Jul 27;18(7):e0288188. doi: 10.1371/journal.pone.0288188 (PMC10374050; doi:10.1371/journal.pone.0288188)
Supplement: Attachment — Submitted filename: CIR review.docx [file pone.0288188.s002.docx]

This is a thorough and well-written analysis of how Citizens’ Initiative Reviews (CIRs) can increase citizens’ knowledge of policy issues prior to voting in a ballot initiative. The authors have a large and good quality dataset which they analyze effectively. This article makes an important contribution to the literature and will, in my opinion, be well cited. I have only a few smaller points that can be addressed through a minor review.

Theory/ framing/ concepts

- From the outset, the article is framed around the problem of growing polarization and misinformation. However, is polarization really the problem that CIRs address? It seems more like the problem they address is a lack of awareness/ lack of opinion (not the problem of strongly held, polarized, partisan beliefs). If the problem is polarization, wouldn’t the solution be moving people toward some kind of more ambivalent position? Learning about the CIR increases both knowledge and confidence of one’s knowledge, which seems like the opposite of ambivalence. I am not saying that increasing confidence of one’s knowledge is a bad thing (increasing confidence of correct knowledge is, as the author’s point out, a good thing). Rather, I think this framing is simply mapping a solution to the wrong problem. This is a more classical epistemic problem of democracy: most people aren’t policy experts/ don’t know much about politics (and have little confidence in what they do know). The CIRs help people overcome this knowledge deficit so that they can make more informed (and confident) decisions about the things that affect them.
- Note that the authors talk about deliberation throughout the paper. It’s even a key moderating variable in the analysis. But deliberation is never defined for the reader. This term should be defined the first time it’s used. Also, more clarity on how respondents’ “faith in deliberation” is measured would be beneficial.
- I find it incredibly interesting that “the CIR’s largest boost was in the proportion of respondents who were confident in their knowledge when their belief was accurate.” This is a novel and important finding. But theoretically, it does not seem to me that increasing one’s confidence in one’s knowledge is the same as increasing knowledge itself. But here knowledge and confidence in knowledge are measured with a single outcome. I could be convinced that this is fine, but I’d like a little more background/theoretical justification for capturing what (at least at first blush) seem to be two concepts with a single outcome.
- P. 10: "In conclusion, this study shows that small scale deliberative interventions can lead to widespread knowledge gains." I'm not sure this conclusion can quite be drawn, at least not without a caveat: the respondents in this study were given the information and were incentivized to read it (they were paid respondents). In the real world, it's not clear how many voters will actually read the CIR recommendation, unprompted. If the statement goes unread, the gains go unrealized. This should be mentioned somewhere.

Methods/analysis/ results

- P. 6 “About 6 percent more of respondents pick the correct answer on individual knowledge items…” Is this difference significant? Should be reported in the text.
- Also, please present the standard error of the mean difference in parentheses in the body of the text.
- P.8 “An additional three cases fit this pattern at a lower level of statistical significance (p < .10).” No, this is not how p-values work. Your alpha (level of significance) is an arbitrary threshold decided in advance. Unless an alpha of 0.10 was specified in advance, just use the default of 0.05. Which means anything greater than 0.05 is simply not significant.

Grammar/Style

- Define acronyms the first time they appear (i.e., Line 3 p. 45 RAND corporation)
- P. 4 line 77/78 there’s a problem with this sentence: "thought one mail survey used a registered voter list and another used a 78 registered voter list."
