# Supplementary material for: Deliberative panels as a source of public knowledge: A large-sample test of the Citizens’ Initiative Review
Source: PLoS One. 2023 Jul 27;18(7):e0288188. doi: 10.1371/journal.pone.0288188 (PMC10374050; doi:10.1371/journal.pone.0288188)
Supplement: Attachment — Submitted filename: Gastil - Reponse to Reviewers.docx [file pone.0288188.s003.docx]

DATE:	June 6, 2023

TO:	PLOS ONE

FROM:	John Gastil, Penn State University, and co-authors

RE:	Revision of PONE-D-23-06764, “Deliberative panels as a source of public knowledge: A large-sample test of the Citizens’ Initiative Review”

# Responses to the Editor’s Letter

Below, we respond to each of the issues emphasized in the editor’s letter.

## Random Selection

**1. “P. 68, you refer to randomly-selected representative bodies. But my understand is that it these samples are not random and include only registered voters. Correct? If so, please clarify.”**

For consistency, we have clarified this point in both places it appears.

## Experts in the CIR

**2. “You refer to experts that will inform citizens. Can you please provide some information about the kind of people that they are. I am not calling for a discussion about the definition of an ‘expert’ but simply some descriptive information.”**

The abstract still refers simply to “neutral experts” because we figured this detail is better elaborated in the manuscript. A sentence was added at the appropriate place.

## Multiple Hypotheses

**3. “On the 82 tests when you unpack the knowledge items. Why did you not correct for multiple hypothesis testing?”**

As we understand it, multiple hypotheses correction is traditionally used when scholars test multiple *qualitatively alternative* hypotheses simultaneously and commonly using the same dataset. Each test has different dependent or independent variables. In that case the risk of committing a Type I error and incorrectly rejecting a single null hypothesis can be high, and the correction discourages authors from proliferating distinct hypotheses in hopes of finding support for one prediction or another. In our case, by contrast, we test consistent hypotheses using the same dependent and independent variables across different knowledge claims, places, and times (scope conditions). As a result, our analytical strategy is to describe the consistency of results across multiple tests.

In our revised version, we decided to a) use Holm multiple hypothesis correction, b) limit our main analysis to reasonably or highly powered Knowledge Items, and c) spell out our analytical strategy more clearly. The updated results are comparable and support the same claims we made in the original submission. Nevertheless, we feel the revised approach is clearer and methodologically stronger. This led to the substantial revision of the results section, “Partisan Bias versus Accuracy Motivation.”

## Project Funding

**4. “We note that the grant information you provided in the ‘Funding Information’ and ‘Financial Disclosure’ sections do not match. When you resubmit, please ensure that you provide the correct grant numbers for the awards you received for your study in the ‘Funding Information’ section.” And later, “Please state what role the funders took in the study. If the funders had no role, please state: ‘The funders had no role in study design, data collection and analysis, decision to publish, or preparation of the manuscript.’ If this statement is not correct you must amend it as needed.”**

As to the role of funders, we can confirm that, “The funders had no role in study design, data collection and analysis, decision to publish, or preparation of the manuscript.”

Our financial disclosure is as follows, and to the best of my knowledge, it matches the Financial Information section at the manuscript submission site. The only tricky point might be that one of the NSF grants was a collaborative proposal, which meant that the budget was split between two universities, each of which got its own NSF award number (1357276 for Gastil and 1357444 for Knobloch). That’s listed in the disclosure as a single grant with the appropriate subtitle, “Collaborative research.” On the Financial Information, we could only log one grant number per entry, so we split it into two entries, one for each Collaborator.

The only other difference we could imagine is the ordering of the grants/contracts. Below, they’re in chronological order. If you’d like the Financial Information rearranged in this way, we weren’t sure how to do that in the submission site.

As for grant numbers, only the NSF grants had meaningful grant numbers associated with them. The rest all had identifying titles, which are provided below.

- “Gastil, J. (2015). Principal Investigator, The Democracy Fund. “2015-2016 Citizens’ Initiative Review Study and Reporting.” ($75,000) [https://democracyfund.org/](https://nam10.safelinks.protection.outlook.com/?url=https%3A%2F%2Furldefense.com%2Fv3%2F__https%3A%2Fdemocracyfund.org%2F__%3B!!OToaGQ!rGZ8PKmqJNXY0tWFny0cEACQjloHZaJZElPrBUHGonPkOQCpcX-oPyj_56Hc3078VoyZ2U3b82pecwhV%24&data=05%7C01%7Cjwg22%40psu.edu%7Ca9d62f25b17c494f03fb08db4a437881%7C7cf48d453ddb4389a9c1c115526eb52e%7C0%7C0%7C638185427047529656%7CUnknown%7CTWFpbGZsb3d8eyJWIjoiMC4wLjAwMDAiLCJQIjoiV2luMzIiLCJBTiI6Ik1haWwiLCJXVCI6Mn0%3D%7C3000%7C%7C%7C&sdata=RPiu0vm4jWdGXFUW5HiHaVJtI1NqGPhzxQchuwzOHG0%3D&reserved=0)
- Gastil, J., & Knobloch, K. (2014). Co-Principal Investigators, National Science Foundation (Directorate for Social, Behavioral and Economic Sciences: Decision, Risk and Management Sciences, NSF Award #1357276/1357444). “Collaborative research: A multi-state investigation of small group and mass public decision making on fiscal and scientific controversies through the Citizens’ Initiative Review.” ($418,000) [https://www.nsf.gov/](https://nam10.safelinks.protection.outlook.com/?url=https%3A%2F%2Furldefense.com%2Fv3%2F__https%3A%2Fwww.nsf.gov%2F__%3B!!OToaGQ!rGZ8PKmqJNXY0tWFny0cEACQjloHZaJZElPrBUHGonPkOQCpcX-oPyj_56Hc3078VoyZ2U3b80DX0XTS%24&data=05%7C01%7Cjwg22%40psu.edu%7Ca9d62f25b17c494f03fb08db4a437881%7C7cf48d453ddb4389a9c1c115526eb52e%7C0%7C0%7C638185427047529656%7CUnknown%7CTWFpbGZsb3d8eyJWIjoiMC4wLjAwMDAiLCJQIjoiV2luMzIiLCJBTiI6Ik1haWwiLCJXVCI6Mn0%3D%7C3000%7C%7C%7C&sdata=FBRTr4KwFi5Zea3JC9Jst%2FKR9pWDqGCXGGeziIqqWcE%3D&reserved=0)
- Gastil, J. (2013). Pennsylvania State University Social Science Research Institute. Award for summer workshop bringing together researchers investigating the Oregon Citizens’ Initiative Review ($5,000) [https://ssri.psu.edu/](https://nam10.safelinks.protection.outlook.com/?url=https%3A%2F%2Furldefense.com%2Fv3%2F__https%3A%2Fssri.psu.edu%2F__%3B!!OToaGQ!rGZ8PKmqJNXY0tWFny0cEACQjloHZaJZElPrBUHGonPkOQCpcX-oPyj_56Hc3078VoyZ2U3b81ImKGiq%24&data=05%7C01%7Cjwg22%40psu.edu%7Ca9d62f25b17c494f03fb08db4a437881%7C7cf48d453ddb4389a9c1c115526eb52e%7C0%7C0%7C638185427047529656%7CUnknown%7CTWFpbGZsb3d8eyJWIjoiMC4wLjAwMDAiLCJQIjoiV2luMzIiLCJBTiI6Ik1haWwiLCJXVCI6Mn0%3D%7C3000%7C%7C%7C&sdata=5RWKZ4F%2BYiVAGqDzIKKSJarmxcBsdJEsrUje%2BuM3aOQ%3D&reserved=0)
- Gastil, J., & Knobloch, K. (2012). Joint learning agreement (research contract) with the Kettering Foundation, with 76% of the budget allocated to Pennsylvania State University and 24% to Colorado State University. “Examining deliberation and the cultivation of public engagement at the 2012 Oregon Citizens’ Initiative Review” ($30,000) [https://www.kettering.org/](https://nam10.safelinks.protection.outlook.com/?url=https%3A%2F%2Furldefense.com%2Fv3%2F__https%3A%2Fwww.kettering.org%2F__%3B!!OToaGQ!rGZ8PKmqJNXY0tWFny0cEACQjloHZaJZElPrBUHGonPkOQCpcX-oPyj_56Hc3078VoyZ2U3b8ypinchz%24&data=05%7C01%7Cjwg22%40psu.edu%7Ca9d62f25b17c494f03fb08db4a437881%7C7cf48d453ddb4389a9c1c115526eb52e%7C0%7C0%7C638185427047529656%7CUnknown%7CTWFpbGZsb3d8eyJWIjoiMC4wLjAwMDAiLCJQIjoiV2luMzIiLCJBTiI6Ik1haWwiLCJXVCI6Mn0%3D%7C3000%7C%7C%7C&sdata=kcjC2BohkQRc1W1cCsut9C3BlWZDVgrJG%2FKl6PlvVG4%3D&reserved=0)
- Gastil, J. (2010). Principal Investigator, National Science Foundation (Directorate for Social, Behavioral and Economic Sciences: Decision, Risk and Management Sciences and Political Science Programs, NSF Award # 0961774), “Investigating the Electoral Impact and Deliberation of the Oregon Citizens’ Initiative Review” ($218,000) [https://www.nsf.gov/](https://nam10.safelinks.protection.outlook.com/?url=https%3A%2F%2Furldefense.com%2Fv3%2F__https%3A%2Fwww.nsf.gov%2F__%3B!!OToaGQ!rGZ8PKmqJNXY0tWFny0cEACQjloHZaJZElPrBUHGonPkOQCpcX-oPyj_56Hc3078VoyZ2U3b80DX0XTS%24&data=05%7C01%7Cjwg22%40psu.edu%7Ca9d62f25b17c494f03fb08db4a437881%7C7cf48d453ddb4389a9c1c115526eb52e%7C0%7C0%7C638185427047529656%7CUnknown%7CTWFpbGZsb3d8eyJWIjoiMC4wLjAwMDAiLCJQIjoiV2luMzIiLCJBTiI6Ik1haWwiLCJXVCI6Mn0%3D%7C3000%7C%7C%7C&sdata=FBRTr4KwFi5Zea3JC9Jst%2FKR9pWDqGCXGGeziIqqWcE%3D&reserved=0)
- Gastil, J. (2010). Principal Investigator, University of Washington Royalty Research Fund. “Panel Survey Investigation of the Oregon Citizen Initiative Review” ($40,000) [https://www.washington.edu/research/or/royalty-research-fund-rrf/”](https://nam10.safelinks.protection.outlook.com/?url=https%3A%2F%2Furldefense.com%2Fv3%2F__https%3A%2Fwww.washington.edu%2Fresearch%2For%2Froyalty-research-fund-rrf%2F*22__%3BJQ!!OToaGQ!rGZ8PKmqJNXY0tWFny0cEACQjloHZaJZElPrBUHGonPkOQCpcX-oPyj_56Hc3078VoyZ2U3b8wEmQydz%24&data=05%7C01%7Cjwg22%40psu.edu%7Ca9d62f25b17c494f03fb08db4a437881%7C7cf48d453ddb4389a9c1c115526eb52e%7C0%7C0%7C638185427047529656%7CUnknown%7CTWFpbGZsb3d8eyJWIjoiMC4wLjAwMDAiLCJQIjoiV2luMzIiLCJBTiI6Ik1haWwiLCJXVCI6Mn0%3D%7C3000%7C%7C%7C&sdata=T75GAvEfcwz63KzwBf%2F6hHaRG9PDRmTTey6Q3t9R58M%3D&reserved=0)

Regarding the Acknowledgements Section, you wrote, “funding information should not appear in the Acknowledgments section or other areas of your manuscript.” We have deleted that information, such that only the first sentence from the Acknowledgement remains.

## Repository

**5. “We note that you have stated that you will provide repository information for your data at acceptance. Should your manuscript be accepted for publication, we will hold it until you provide the relevant accession numbers or DOIs necessary to access your data…”**

Once the paper is accepted, we’ll post our data and code on Harvard Dataverse (or comparable website) to publish the finalized data and analysis code.

## IRB/Ethics statement

**6. “Please include your full ethics statement in the ‘Methods’ section of your manuscript file. In your statement, please include the full name of the IRB or ethics committee who approved or waived your study, as well as whether or not you obtained informed written or verbal consent. If consent was waived for your study, please include this information in your statement as well.”**

Following this advice, we simplified the ethics statement, which now matches our Methods section.

## Supporting Information and References

**7. “Please include captions for your Supporting Information files at the end of your manuscript, and update any in-text citations to match accordingly. Please review your reference list to ensure that it is complete and correct.”**

We have done so.

# Responses to Reviewer 1

The first reviewer offered a very favorable assessment but requested clarifications.

## Sampling Periods

**1. “First, do the sampling periods correspond to the periods before the actual ballot measures? Are respondents asked about their exposure to, say, voter guides that contain the statements used in the survey?”**

We added this sentence to clarify these details.

## Confidence in Deliberation

**2. “Second, the question of respondents’ “confidence in the deliberative process and the CIR is a bit ambiguous (177ff). One can have confidence that ‘ordinary citizens’ will learn and make good judgments. Or one can have confidence in the deliberative processes. The first bit of information is easier for citizens to gain; the second requires a bit more effort.”**

The easiest way to clarify this was to include the exact phrasing of the three survey items that make up this “confidence in deliberation” scale. We did so in a footnote, then paraphrased the items more succinctly in the text.

## Dataset Uniqueness

**3. “Third, the statement that this survey dataset ‘has permitted a fine-grained analysis not seen before in this field of study’ is quite right for CIR processes. However, there is at least one other such dataset in the broader field of democratic innovations, that collected by Jim Fishkin and his colleagues on Deliberative Polling, which also involves a broad range of consistent questions aggregated over many dozens of events, all of which have close to the same structure.”**

We rephrased for clarity. The Deliberative Polling data collected by Fishkin repeats measures of the pre- and post-deliberations of the participants in the minipublic. (The Participedia database is somewhat similar, if more diffuse in what gets measured about the minipublics included therein.) We meant to emphasize the value of having repeated measures of a minipublic’s wider impact on the public, or in this case more specifically the “electorate,” across multiple issues. Thus, we rephrased accordingly.

# Responses to Reviewer 2

The second reviewer gave a very favorable assessment but requested clarifications and changes, some of which have been addressed already in this response.

## Polarization Framing

**1. “From the outset, the article is framed around the problem of growing polarization and misinformation. However, is polarization really the problem that CIRs address? It seems more like the problem CIRs address is a lack of awareness/ lack of opinion (not the problem of strongly held, polarized, partisan beliefs).”**

We were unsure where the manuscript foregrounded the problem of polarization. We refer to a “public divided over objective facts featured in policy debates” (abstract) and open the introduction by warning of an “epidemic of ‘truth decay’,” but these concern underinformed and misinformed voters, not affective/ideological polarization, per se. We discuss the CIR’s epistemic value right through to the last sentence, “In a world beset by misinformation and misperception…, such a body of knowledge could not be more timely.”

Perhaps the problem was leading with how contemporary politics and media skew voters’ factual beliefs by appealing to their preexisting biases. To address this, we found a way to make this epistemic frame clearer in the opening sentence. We moved up one of our references (and renumbered accordingly) toward this end.

## Define Deliberation

**2. “…the authors talk about deliberation throughout the paper. It’s even a key moderating variable in the analysis [faith in delib]. But deliberation is never defined for the reader. This term should be defined the first time it’s used.”**

We addressed this oversight by using a succinct and useful definition

As noted earlier in this response, we also clarified the meaning of the “faith in deliberation” scale we use in the study. This included listing all three survey items in a footnote.

## Accuracy and Confidence

**3. “I find it incredibly interesting that ‘the CIR’s largest boost was in the proportion of respondents who were confident in their knowledge when their belief was accurate.’ This is a novel and important finding. But theoretically, it does not seem to me that increasing one’s confidence in one’s knowledge is the same as increasing knowledge itself.”**

If we had pre- and post-CIR data, we could delve deeper into this finding and make more of it, as the reviewer suggests. Alas, we can’t know how individual respondents are moving on this continuum from confident-and-incorrect to confident-and-correct. We can only see the aggregate movement in this regard. Moreover, we don’t have these questions branched in every survey (i.e., asking first if a claim is true/false, then asking for a confidence judgment).

What we can do, however, is suggest future research that can disentangle these effects better. Our penultimate paragraph now reads:

## Widespread Gains

**4. “In conclusion, this study shows that small scale deliberative interventions can lead to widespread knowledge gains.” I’m not sure this conclusion can quite be drawn, at least not without a caveat: the respondents in this study were given the information and were incentivized to read it (they were paid respondents). In the real world, it’s not clear how many voters will actually read the CIR recommendation, unprompted. If the statement goes unread, the gains go unrealized. This should be mentioned somewhere.”**

Fortunately, the adoption rate is known based on other data, which we now cite in the conclusion to make this point clearer.

## Minor corrections requested

**5. “P. 6 [reads], ‘About 6 percent more of respondents pick the correct answer on individual knowledge items…’ Is this difference significant? Should be reported in the text.”**

The details of those tests appear in the Online Supplement, as noted in the paper.

**6. “Also, please present the standard error of the mean difference in parentheses in the body of the text.”**

We now do this.

**7. “P.8, ‘An additional three cases fit this pattern at a lower level of statistical significance (p < .10).’ No, this is not how p-values work. Your alpha (level of significance) is an arbitrary threshold decided in advance. Unless an alpha of 0.10 was specified in advance, just use the default of 0.05. Which means any p-value greater than 0.05 is simply not significant.”**

We may disagree about how best to treat *p*-values, but we concur with the spirit of this point. This led to the substantial revision of the entire results section entitled “Partisan Bias versus Accuracy Motivation.”

**8. “Define acronyms the first time they appear (i.e., Line 3 p. 45 RAND corporation)”**

RAND is a tricky thing—not quite an acronym but an actual name. Technically, it awkwardly stands for “Research ANd Development,” but the all-caps “RAND” is rarely, if ever, decomposed that way.

The acronyms that do appear (e.g., CIR) are handled as requested.

**9. “P. 4 line 77/78 there’s a problem with this sentence: “thought one mail survey used a registered voter list…”**

The word “thought” was a typo version of “though.” This has been fixed.
